# Draft Genome Sequence of *Desulfocarbo indianensis* SCBM, a New Genus of Sulfate-Reducing Bacteria, Isolated from Water Extracted from an Active Coalbed Methane Gas Well

**DOI:** 10.1128/genomeA.00970-15

**Published:** 2015-09-03

**Authors:** Thuy T. An, Flynn W. Picardal

**Affiliations:** School of Public and Environmental Affairs, Indiana University, Bloomington, Indiana, USA

## Abstract

We used Illumina MiSeq technology to sequence the whole genome of *Desulfocarbo indianensis* SCBM, a new genus of sulfate-reducing bacteria isolated from a coal bed in Indiana, USA. This draft genome represents the first sequenced genome of the genus *Desulfocarbo* and the second known genome of the order *Desulfarculales*.

## GENOME ANNOUNCEMENT

*Desulfocarbo indianensis* SCBM is a vibrio-shaped, polarly flagellated, motile, and strictly anaerobic sulfate-reducing bacterium, isolated from a benzoate-oxidizing and sulfate- and iron-reducing enrichment that was inoculated with water extracted from a coal bed in Indiana, USA (1, 2). It is the type species of the new genus *Desulfocarbo*, which was first described in 2014, and it represents only the second known member of the order *Desulfarculales* after *Desulfarculus baarsii* DSM 2075 (1). It was deposited in three publicly accessible culture collections (accession numbers: DSM 28127, JCM 19826, and ATCC BAA-2625). Strain SCBM is of interest because it is the first sulfate-reducing bacterium retrieved from a coalbed environment and it may play an essential role in anaerobic carbon metabolism in coal beds. To better understand its physiological, metabolic, and genetic traits that allow it to be successful in coalbed environments, the whole genome of strain SCBM was sequenced, assembled, and annotated.

The total genomic DNA of strain SCBM was extracted using an UltraClean microbial DNA isolation kit, and the paired-end library was prepared using a TruSeq DNA library prep kit. The whole genome was sequenced using an Illumina MiSeq sequencer with 320- and 200-bp paired-end runs generating 4,126,300 paired-end reads. Read trimming, base correction, and *de novo* assembly were performed using the A5-MiSeq assembly pipeline version 20141120 (3). The draft genome included 335 contigs in 334 scaffolds with an *N*_50_ and *N*_90_ of 518,820 and 78,602 bp, respectively. The longest and shortest sequences were 1,537,261 and 638 bp, respectively. The total estimated size of the whole genome was 5,114,044 bp (mean depth of coverage, 309X) with a GC content of 63%, a little higher than the GC content determined by the HPLC method (62.5% mol) (1). As assessed by CheckM (4), the completeness and coding density of the genome were 98.08% and 87.64%, respectively. The genome was structurally and functionally annotated using the NCBI Prokaryotic Genome Annotation Pipeline (PGAP) version 2.1 (5) and the Rapid Annotations using Subsystem Technology (RAST) version 2.0 (6, 7). The PGAP identified 4,771 genes, 4,228 coding sequences, 490 pseudogenes, 1 regularly interspaced short palindromic repeat (CRISPR) array, 3 rRNAs (5S, 16S, and 23S), 49 tRNAs, and 1 noncoding RNA (ncRNA). Classic RAST annotation with the Glimmer3 gene caller yielded 4,803 coding sequences, 3 rRNAs (5S, 16S, and 23S), and 48 tRNAs.

As expected from laboratory experiments (1), the genome contains a variety of genes encoding for benzoate and fatty acid degradation, dissimilatory sulfate reduction, and carbon fixation. Moreover, a total of 165 genes involved in motility and chemotaxis are present in the genome. This is consistent with our observation that strain SCBM is a motile bacterium containing a monotrichous polar flagellum (1). Making the genome of strain SCBM available to the public enables more comprehensive and comparative genomic analyses that expand our understanding of anaerobic, coalbed carbon metabolism. A complete whole-genome analysis is in progress with an expectation to reveal additional information on the catabolic potential of *D. indianensis* SCBM.

### Nucleotide sequence accession numbers. 

This whole-genome shotgun project has been deposited at DDBJ/EMBL/GenBank under the accession number LBMP00000000. The version described in this paper is the first version, LBMP01000000.
